# Surpassing 99% detection efficiency by cascading two superconducting nanowires on one waveguide with self-calibration

**DOI:** 10.1038/s41377-025-02031-5

**Published:** 2025-10-17

**Authors:** Zhen-Guo Li, Jun Mao, Yi-Jin Zhou, Jia-Wei Guo, Shi Chen, Hao Hao, Yang-Hui Huang, Sai-Ying Ru, Nai-Tao Liu, Zhen Liu, Jie Deng, Fan Yang, Xue-Cou Tu, La-Bao Zhang, Xiao-Qing Jia, Jian Chen, Lin Kang, Jianwei Wang, Qing-Yuan Zhao, Qihuang Gong, Pei-Heng Wu

**Affiliations:** 1 Research Institute of Superconductor Electronics (RISE), School of Electronic Science and Engineering, Nanjing University, Nanjing, 210023 China; 2 Purple Mountain Laboratory, Nanjing, 211111 China; 3 State Key Laboratory for Mesoscopic Physics, School of Physics, Peking University, Beijing, 100871 China; 4 Hefei National Laboratory, Hefei, 230088 China; 5 Frontiers Science Center for Nano-optoelectronics & Collaborative Innovation Center of Quantum Matter, Peking University, Beijing, 100871 China

**Keywords:** Integrated optics, Quantum optics, Quantum optics

## Abstract

Integrated quantum photonics (IQP) allows for on-chip generation, manipulation and detection of quantum states of light, fostering advancements in quantum communication, quantum computing, and quantum information technologies. Single-photon detector is a key device in IQP that allows for efficient readout of quantum information through the detection of single-photon statistics and measurement of photonic quantum states. The efficacy of quantum information retrieval hinges on the ability to simultaneously detect every single photon with high efficiency, a relationship that grows exponentially with the number of photons (*n*). Even a slight decrease in single photon detection efficiency can lead to a significant reduction in probability as *n* grows larger. Here, we introduce a superconductor-semiconductor heterogeneous integration technology that allows for the integration of transversal superconducting nanowires single-photon detectors that eliminate corner loss on various optical waveguides with exceptional efficiency and versatility. Two cascaded nanowires have been integrated on one silicon waveguide, which not only boosts the detection efficiency to 99.73% at a wavelength of 1550 nm but also provides an on-chip calibration setup, allowing such high efficiency to be measured despite the large loss from fiber-to-waveguide coupling and uncertainties from absolute power calibrations. These advancements represent a substantial improvement compared to previous records, approaching the theoretical limit achievable on silicon waveguide, and demonstrate the versatility of heterogeneous integration technology. This breakthrough in ultra-high detection efficiency establishes a new baseline for assessing quantum measurement capabilities on scalable IQP platforms.

## Abstract (web summary)

Superconducting Nanowires on Silicon Waveguide Achieve 99.73% Single-Photon Detection Efficiency, Enabling Precision Quantum Measurement on Scalable Integrated Platforms.

## Graphical abstract

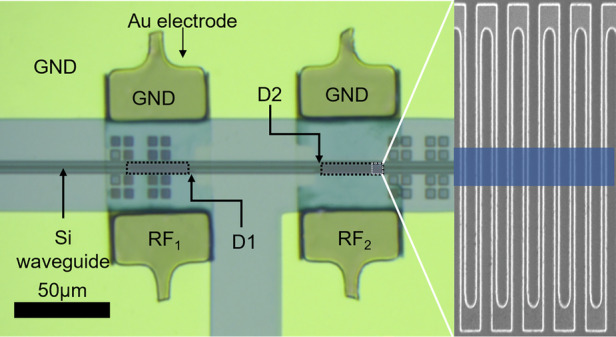

## Introduction

Harnessing single photon as the carrier of quantum information, integrated quantum photonics (IQP) technologies^1^ represent an attractive platform for investigations and implementations of quantum systems at scale. Benefiting from advanced semiconductor manufacturing processes and the low-noise property of photons, scalability is one of the advantages of IQP, which gives great promising for achieving a useful quantum computer of millions of qubits^2^. Single-photon detector (SPD)^3^ is one of the critical elements in an integrated photonic chip as well as single photon sources (SPS)^4^, modulators^5^, optical circuits^6^, and readout circuits^7^. However, these components need to be designed beyond their state-of-the-art performance to meet critical requirements of an IQP^2^. Low detection efficiency^8^ limits the success probability of quantum gate^9^ operations and state measurements^10^, and therefore the scalability of IQP. Therefore, highly efficient waveguide integrated SPDs are required to avoid fiber-to-waveguide loss. Taking Boson sampling task as an example^11^, the probability of measuring the coincidence of 100 photons would be 0.008% considering a detection efficiency of 91%^12^ reported previously at each channel. Although this efficiency is already advanced compared to other commercial SPDs, the resulted coincidence probability is too low for supporting large scale quantum simulations. In comparison, a detection efficiency of 99.9% would increase a 100-photon coincidence detection probability to 90.48% and 1000-photon coincidence detection probability to 36.77%. On the other hand, on-chip detection can minimize the detection delay, benefiting feedforward error correction in a universal photonic computing architecture^13^.

Compared to semiconductor single-photon avalanche diodes (SPADs)^14,15^, low temperature superconducting sensors, where transition edge sensors (TES)^16,17^ and superconducting nanowire single-photon detectors (SNSPDs)^18,19^ are the two most attractive devices, show superior performance in high detection efficiency and low dark counts. Compared to TESs, SNSPDs are thin-film based devices and their feature size is typically less than the width of optical waveguide^20^. Thus, superconducting nanowire can be deposited and patterned on top of a waveguide. Moreover, SNSPDs are faster than TESs, showing higher photon counting rate^21^ and lower timing jitter^22^. The typical operation temperature of SNSPDs is ~2 K while it is ~100 mK for TESs^23^. Thus, considering large-scale integration in future, SNSPDs require less cryogenic resource. These advantages make SNSPDs a promising candidate for building scalable and fast IQPs, which have been demonstrated on various platforms, such as silicon, silicon nitride^24^ and lithium niobate^25^. A near perfect SPD with on-chip detection efficiency (ODE) as close as possible to unity is demanded for a quantum photonic computer containing enormous qubits.

**Fig. 1:**
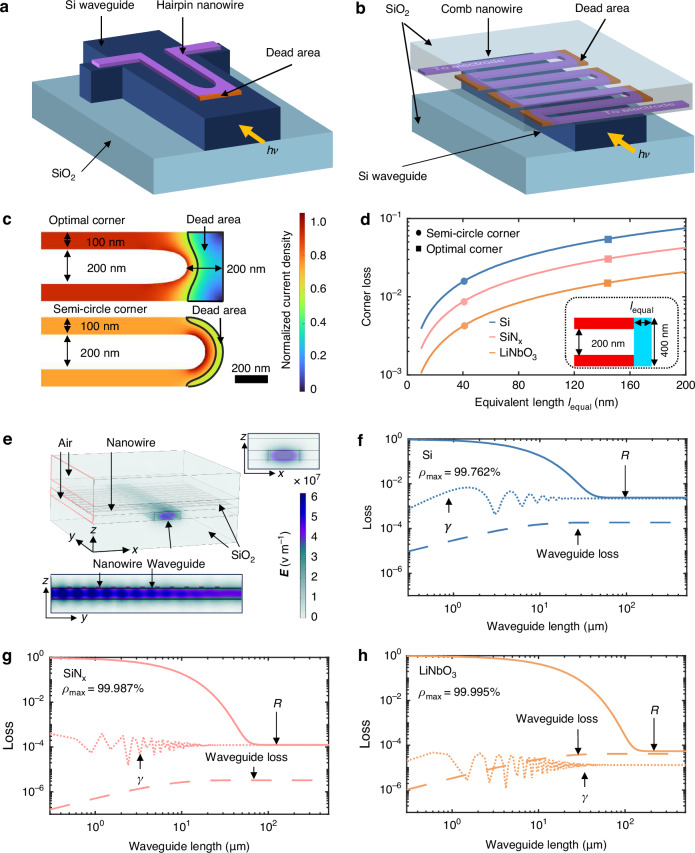
A high efficiency comb nanowire detector design suitable for various waveguides. Conceptual diagrams of a hairpin nanowire structure (**a**) and a comb nanowire structure (**b**). Corners are marked in red. **c** Current density simulations for optimal corner (rectangular outer/streamlined inner edge, 200 nm spacing) and semicircular corner (width 100 nm, radii 200 nm). NbN nanowire parameters: width 100 nm, gap 200 nm, thickness 5 nm. **d** Corner loss vs equivalent length ${l}_{{\rm{equal}}}$ is illustrated, where the definition of equivalent length ${l}_{{\rm{equal}}}$ is presented in the inset figure. The SNSPD is modeled as a combination of an ideal straight-line structure (red region) and a dead area (blue region), with the dead zone width fixed at 400 nm. The equivalent length is derived from both the actual corner geometry and the current density distribution profile. Loss of the corners drawn in (**c**) is marked. **e** 3D simulation of the electric field intensity distribution in the waveguide-integrated comb nanowire, along with the cross-sectional plot in the waveguide propagation direction (y-z plane) and the end-face mode field distribution plot (x-z plane). Simulations of the residual absorption $R=1-p$ of the comb nanowire on Si (**f**), SiNx (**g**) and LiNbO_3_ (**h**) waveguides. $p$ is the net absorption by the nanowire

Here, we propose a transversal alignment of meandered superconducting nanowire instead of a longitudinal alignment on top of a waveguide combined and have developed a heterogeneous semiconductor-superconductor integration method^19^ that is compatible to various popular waveguide platforms. Detector architecture is shown in Fig. 1. Benefiting from the increased enhanced evanescence coupling efficiency, the waveguide span where the superconducting nanowire is place on can be reduced to a shorter distance compared to conventional hairpin structure. Transverse alignment also avoids corners on waveguide so that every absorbed photon can generate a detection pulse, pushing the quantum efficiency of the entire nanowire detector to 100%. In addition, transversal alignment and heterogeneous integration method maintain the propagation mode of the guided light. Thus, by placing two identical detectors on the same waveguide one after another, an in-situ and self-calibration setup can be built on chip. The latter detector absorbs and detects the residual photons left by the first one so that the response ratio between them can be used for extracting the net on-waveguide photon absorption. This calibration method removes the uncertainty of fiber-to-waveguide coupling during on-waveguide efficiency characterization. The proposed method is verified on a silicon waveguide, but can be adapted to other waveguide platforms due to the hybrid integration advantage. Both room-temperature optical transmission measurements and cryogenic photon-counting measurements show consistent results. The successive *ODE* of the two detectors is 99.73%, including the 0.22% reflection loss from the detector to waveguide. This near-unity high efficiency overcomes the bottleneck of detections in an IQP, encouraging implementations of large-scale photonic quantum simulating and computing towards thousands of photons^26^.

## Results

### Simulations of maximum on-chip detection efficiencies on various waveguide platforms

Waveguide integrated SNSPD is typically designed in a hairpin structure as shown in Fig. 1a and lithographically fabricated on the same waveguide chip. The superconducting nanowire has a typical width less than 100 nm, which is narrower than the width of an optical waveguide. The width of waveguide limits the space where nanowires can be placed. Considering the tolerance of alignment errors and the two electric connections on the opposite side of the incident light, it is common to have two nanowires meandered in the center of the waveguide. This structure is typically named as a hairpin structure. To achieve high-performance hairpin SNSPDs, both the waveguide and superconducting nanowire fabrication processes must be optimized, posing challenges in uniform superconducting film deposition and precise pattern alignment. Although the state-of-the-art on-chip detection efficiency of hairpin detectors can exceed 90%^27–33^, further increasing the efficiency to approach 100% requires addressing minor loss factors that are often overlooked.

First, the corner in front appears as a limiting factor for achieving near-unity detection efficiency compared to waveguide loss and reflection loss. In typical meandered nanowire detector, the corner is designed into a rectangle with an optimal curve on the inner side, as shown in Fig. 1c, to avoid current crowding so that the nanowire can be biased higher for achieving well saturated quantum efficiency^34^. Current distributed around the outer corner is much lower than the crucial current so that this area is insensitive to photons and becomes a dead area. To minimize such loss, hairpin detectors also use semi-circular corners as shown in Fig. 1c. However, current crowding effect around the corner limits the maximum bias current of the longitudinal nanowires. Furthermore, the corner of a hairpin nanowire detector is positioned at the front while its electrical leads are at the back. Thus, although the equivalent length of the dead area is short, the incident photon encounters this corner first, resulting in a significant loss compared to other minor loss factors. From the numerical calculations as shown in Fig. 1d, the optimal corner exhibits an equivalent length ${l}_{{\rm{equal}}}$ of 139 nm, corresponding to a propagation loss of 5.29% in silicon waveguide. The semicircle configuration demonstrates an equivalent length of 49 nm with a lower propagation loss of 1.90% in silicon waveguide. The detailed calculation procedure is provided in the Supplementary Materials. An alternative and intuitive solution for avoiding the corner loss is to place nanowires transversely, as shown in Fig. 1b. We name this the comb structure for clarification.

While a long nanowire can have higher absorption, the waveguide underneath can dissipate photons as well. The comb structure also shows advantage in higher absorption coefficient so that short waveguide length is required. For waveguide detectors, the nanowire absorbs the evanescence light leaked outside of the waveguide. The absorption coefficient per unit length depends on the optical properties, location of the nanowires, and mostly important, the portion of the nanowire volume to the guided optical mode volume. Comb nanowire can cover the entire top surface of the waveguide. Its absorption coefficient ($7.57\times {10}^{3}{\rm{dB}}/{\rm{cm}}$ on Si waveguide, $7.29\times {10}^{3}{\rm{dB}}/{\rm{cm}}$ on SiNx waveguide, and $3.59\times {10}^{3}{\rm{dB}}/{\rm{cm}}$ on LiNbO_3_) is higher than that of a hairpin nanowire ($6.06\times {10}^{3}{\rm{dB}}/{\rm{cm}}$ on Si waveguide, $2.55\times {10}^{3}{\rm{dB}}/{\rm{cm}}$ on SiNx waveguide, and $1.62\times {10}^{3}{\rm{dB}}/{\rm{cm}}$ on LiNbO_3_) even considering the filling factor. A short waveguide where the nanowire is placed on reduces the waveguide loss, in particular for waveguides of large mode size. For example, in LiNbO_3_ waveguide, a hairpin nanowire requires a waveguide length of 123.3 μm for achieving 99% absorption, while the comb nanowire only requires 54.6 μm.

After removing the corner loss by using the comb structure, reflection loss becomes another factor that is small but considerable. The large over area of the comb nanowire increases the absorption coefficient but also introduces a relatively larger reflection than the hairpin detector. The comb nanowire functions as a lossy optical grating. As shown in the numerical simulation in Fig. 1f–h, interference patterns are observed with regards to the number of transverse nanowire segments on top of the waveguide. After several oscillations, reflection coefficient goes to saturate at $2.19\times {10}^{-3}$ for Si waveguide, $1.21\times {10}^{-4}$ for SiNx waveguide and $1.32\times {10}^{-5}$ for LiNbO_3_ waveguide. Light scattering out of the waveguide induced by the comb nanowire should be less than the reflection due to the weak evanescence coupling. In our comb nanowire detector, the membrane substrate is a 256 nm thick SiO_2_, which is typically used as the cladding material on top of waveguide due to its low refractive index. The nanowire is several nanometers thick. From a 3D numerical simulation as shown in Fig. 1e, light is tightly confined in the waveguide and its power density exponentially decays due to the absorption of the nanowire. After integration, the real part of the mode index varies from 2.8294 of a bare Si waveguide to 2.8418.

When the above loss factors are all considered, the ultimate on-waveguide detection efficiency of the comb nanowire can be calculated. The calculation details are given in the supplementary material, verified both by 3D simulation and transmission matrix method. The maximum nanowire absorption ${p}_{\max }$ is 99.762% for Si waveguide, 99.987% for SiNx waveguide and 99.995% for LiNbO_3_ waveguide. The on-waveguide detection efficiency *ODE* = $\eta \cdot {p}_{\max }$ is the product of the quantum efficiency of the nanowire $\eta$ and ${p}_{\max }$.

### Precise hybrid integration using a PDMS stamp

The comb nanowire detector is attractive but faces challenges in lithography. It has been tried to fabricate along with waveguide, forming a sub-wavelength multi-mode interference structure^12^. The meander structure was transferred both into the superconducting film and the underlying substrate. The discontinuous waveguide had a very high loss and the nanowire showed a low detection efficiency. An alternative approach is depositing cladding material on waveguides and then polishing the surface for the following superconducting film deposition and nanowire patterning. This process is possible but requires advanced manufactural equipment and process^2^. The polishing process needs to guarantee very low surface roughness, which is crucial for depositing high-quality superconducting films. The top surface of the waveguide needs to be exposed after polishing so that the polishing depth needs to be controlled precisely. Discontinuity of the waveguide material and cladding material underneath the film may create artificial grain boundaries that suppress the switching current of the patterned nanowire.

**Fig. 2:**
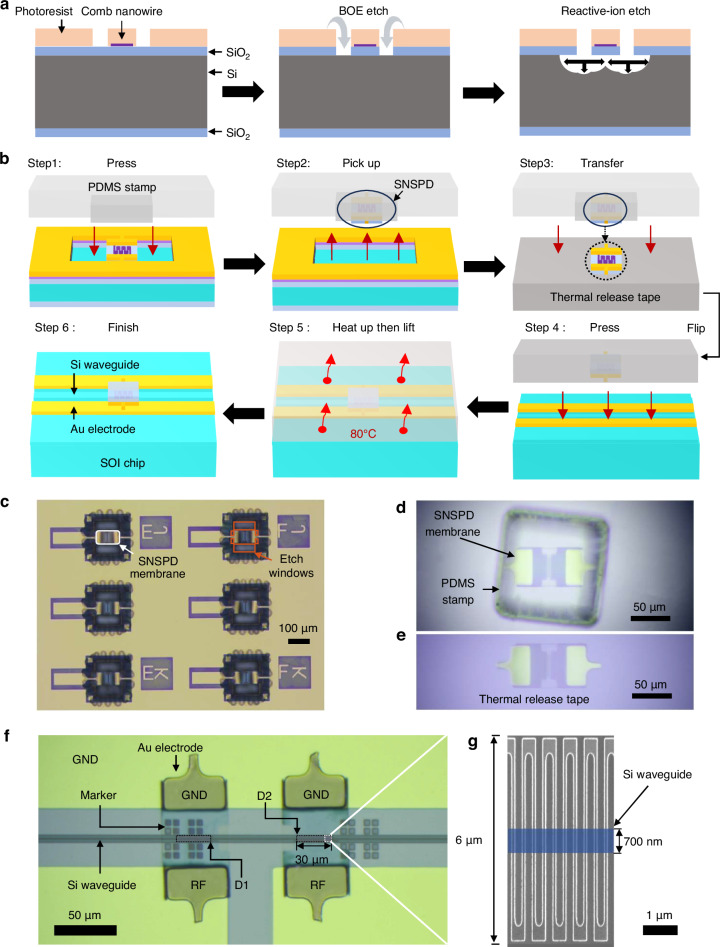
Hybrid integration of two comb nanowire detectors on one optical waveguide. **a** Etching processes for the suspended SNSPD membranes are presented. It should be noted that the figures are not drawn to scale with respect to the actual device dimensions. **b** Diagrams showing the processes of hybrid transferring comb nanowire detectors onto waveguides. **c** Microscope image of the chip where the big pads are for testing in a probe station and the central detectors are etched into membranes. **d** Optical microscope image of a comb nanowire detector on the PDMS stamp. **e** Optical microscope image of a comb nanowire detector on the thermal release tape. **f** An optical microscope image of two comb nanowire detectors placed on the same silicon waveguide. **g** The inset scanning electron microscope image shows a portion of the meandered nanowire. The blue rectangle indicates the location of the silicon waveguide underneath. The nanowire is 100 nm wide with a spacing of 200 nm

Hybrid integration offers a promising solution to overcome the fabrication difficulties of comb structure nanowires on waveguides. This has been first demonstrated by Faraz et al.^27^ except that the nanowire placed on the waveguide is designed in a longitudinal meandered structure that includes corner loss. Here, we refer to the hybrid integration method to place a comb structure nanowire on a Si waveguide. Additionally, to have robust and precise picking and placing steps, an upgraded hybrid integration process is developed, which is shown in Fig. 2b and details are given in the method section. In brief, nanowire detectors were fabricated on thermally oxidized double-side polished silicon wafers with a 256 nm SiO₂ layer. After defining a photoresist mask, BOE (Buffered Oxide Etchant) was used to open the oxide windows, followed by anisotropic SF₆-based RIE (Reactive Ion Etching) to etch the underlying silicon and release the suspended membranes, as shown in Fig. 2a, c. The SiO_2_ membrane formed a flexible substrate and a cladding on top of waveguide after transfer. The nanowire, as shown in Fig. 2g, was designed into 100 nm wide and 200 nm gap, giving a filling factor of $1/3$. It was meandered into a rectangle area. The width was 6 μm and the length was 30 μm. A PDMS post was used to pick the membrane and place on a thermal-released tape, as shown in Fig. 2b, d. This step also flipped the chip upside down so that the electrical pads on the membrane chip can be contacted with the complementary pads on the host waveguide chip. Then, the membrane was aligned and placed on the waveguide chip. After heating the release tape and lifting it with a controllable speed, the membrane detectors were left on the waveguide chip via van der Waals force, as shown in Fig. 2f. Although a 200 nm height difference existed at the gold layer, this variation was relatively small compared to the device dimensions of 50 μm × 100 μm. The flexible membrane can make sufficient contact with the waveguide and pads to ensure structural and electrical integrity. In our measurement setup, the integrated chip was mounted on the cold plate upside down through multiple thermal cycling. Based on our experience, the contact strength was sufficient to hold the membrane chip during integration and testing. Total contact resistance was less than 10 Ω. The planar stamp technique proved more reliable in operation compared to conventional tungsten tip approaches. Since the membrane substrate was transparent, optical waveguide and comb nanowire detector can be observed under an optical microscope at $500\times$ magnification, achieving micrometer alignment resolution. Simulation results showed that the absorption was insensitive to tilting under this resolution. The detailed simulation methodology is provided in the Supplementary Information.

The hybrid method separates the fabrication of detectors and optical waveguides. The chip was first characterized under a customized 1.5 K probe station for screening detectors of saturated quantum efficiency and then etched into membrane detectors. A typical fabrication yield in our university cleanroom was around 80% (Statistical data are given in the supplementary materials). The microchip transfer process is robust and reproducible. Moreover, the method is compatible with flip-chip integration process in semiconductor electronics and could be done automatically using commercial assembly equipment. Figure 2f shows an example of two detectors integrated on a single waveguide. Their switching currents were 6.46 μA and 6.44 μA for D1 and D2. Moreover, these two devices exhibited saturated quantum efficiency. Thus, they can be taken as identical devices.

### A relative calibration method removing uncertainties from absolute incident power

The conventional method of characterizing the detection efficiency is based on measuring the ratio between the number of photon detection pulses and the calibrated incident photon number. However, the number of incident photons is difficult to calibrate precisely on waveguide due to a large uncertainty from fiber to waveguide coupling loss, variation between the calibrated couplers and the one used for detectors, and accuracy of commercial power meters^3^. In our cryogenic setup, the system detection efficiency measured from the incident fiber to the on-waveguide detector was 14.3% (data are given in the supplementary information), where most loss was caused by the weak fiber-to-waveguide coupling efficiency. Under such high coupling loss, a precise calibration of the *ODE* was difficult. Moreover, the uncertainty of the calibrated power meter typically ranges from 2% to 5%, which limits the accurate calibration of detectors with efficiencies higher than the precision of the instrument. The hybrid integration process enables two membrane detectors to be placed on a single waveguide, offering a relative method for calibrating *ODE*. The relative method is based on successive absorption and detection of the travelling light so that the absolute input photon flux ${N}_{{\rm{in}}}$ cancels out after calculation.

**Fig. 3:**
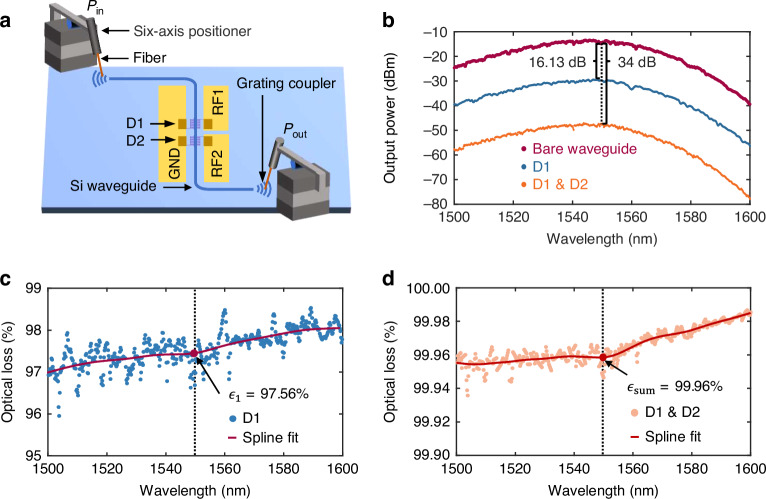
Light absorption measurement at room temperature **a** A conceptual diagram of the measurement setup. **b** Measured output power of the bare waveguide, after integration of one detector (D1), and two detectors (D1 & D2). These measurements are conducted on the same waveguide chip step by step. Calculated optical loss caused by D1 (**c**) and by D1 & D2 (**d**) in percentage

First, the successive absorption of light was verified by in-situ optical transmission measurements at room temperature. As shown in Fig. 3a, a fiber-to-waveguide-to-fiber coupling setup was used to measure the absorption of the comb nanowire detector before and after hybrid integration for the same waveguide. After the first detector was placed on, there was a 16.13 dB reduction in transmission at the wavelength of 1550 nm, which corresponds to an optical loss of ${\epsilon }_{1}=97.56 \%$. After placing the second detector, the total optical loss of the waveguide with two detectors was ${\epsilon }_{{\rm{sum}}}=34.0\,{\rm{dB}}=99.96 \%$. In this case, the two detectors can be taken as a single extended detector for achieving higher absorption, although their detection counts can be read out individually. The waveguide loss was cancelled out by subtracting the transmission of the bare waveguide and its value was smaller than the reflection by one order of magnitude from the simulation shown in Fig. 1f. Thus, the optical loss was the sum of the absorption of the comb nanowire detector and the reflection caused by placing the detector on. Considering the reflection of the incident light, which saturated at $\gamma =0.22 \%$ based on simulation, the net absorption of D1 and D1&D2 were ${p}_{1}={\epsilon }_{1}-\gamma =97.34 \%$ and ${p}_{{\rm{sum}}}={\epsilon }_{{\rm{sum}}}-\gamma =99.74 \%$, respectively.

This in-situ room temperature measurement of the absorption avoided uncertainties among calibrations of the incident light power, grating coupler transmission and waveguide loss on different chips. The same waveguide chip was measured at different configurations. Moreover, the absorption measurement can be done with much higher incident power than single-photon counting measurement at cryogenic temperature. Therefore, the power meter exhibited better accuracy and linearity. Since absorption was extracted from the relative loss, DC offset errors from the power meter can also be reduced.

**Fig. 4:**
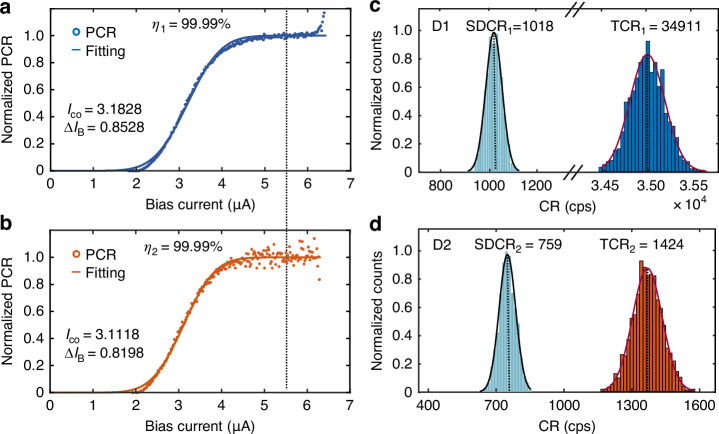
Light absorption measurement at cryogenic temperature using relative photon counts Normalized PCR versus bias current curves in linear scale for D1 (**a**) and D2 (**b**). Lines are fitting curves using sigmoidal functions. Statistical distributions of the SDCR and TCR for D1 (**c**) and D2 (**d**) at a constant bias current of 5.5 ${\rm{\mu }}{\rm{A}}$. Lines are fitting curves using Gaussian functions

In principle, if the comb detector exhibited a near-unity quantum efficiency, the *ODE* of the detector can be calculated by *ODE* = $\eta \cdot p$. Direct measurement of the quantum efficiency of SNSPDs inherits problems of uncertainty during calibrating the incident photon number. An alternative way is extracting the quantum efficiency from the dependence of photon counting rate on bias current. As shown in Fig. 4c, d, the normalized photon counting rate (PCR) curves of both detectors show well saturation. Based on the Fano fluctuation model^35^, the photon detection goes into deterministic regime as the bias current increases, where every absorbed photon can register a detection pulse, namely having a quantum efficiency of 100%. The normalized PCR curve can be fitted by a sigmoidal curve $\eta =\frac{1}{2}{erfc}(\frac{{I}_{{\rm{co}}}-{I}_{{\rm{B}}}}{\Delta {I}_{{\rm{B}}}})$^35^ based on this assumption. Fitting parameters ${I}_{{co}}$ and $\sigma$ are shown in Fig. 4a, b. By substituting the bias current ${I}_{B}$ into the equation, the corresponding quantum efficiency $\eta$ of the device can be calculated. At a bias current of 5.5 μA, the quantum efficiencies for both detectors can reach ${\eta }_{1}={\eta }_{2}={\eta }_{0}=99.99 \%$. Details of the fitting and confidence interval are given in the supplementary information. By taking into account the absorption ratio, on-chip detection efficiencies for D1 and D1 & D2 are ${OD}{E}_{1}={\eta }_{0}\cdot {p}_{1}=97.33 \%$, and ${OD}{E}_{{\rm{sum}}}={\eta }_{0}\cdot {p}_{\rm{sum}}=99.73 \%$.

The above calculation of the *ODE* was based on room temperature measurement of the absorption and the cryogenic measurement of the quantum efficiency. One remaining question would be these two measurements were done at different temperatures so that the true absorption when the detectors were superconducting may vary. To address this concern, the successive architecture of the two detectors on one waveguide can also offer a relative calibration based on their photon counting rates. Compared to the room temperature transmission measurement setup, the cryogenic measurement took the two identical detectors as on-chip power meters for self-calibration. The absolute photon counting rate of D2 was much lower than that of D1, proving the validity of successive absorption and detection at cryogenic temperatures. Photon counting rates for D1 and D2 can be written as:

1 $${{PCR}}_{1}={{N}_{{\rm{in}}}\cdot \eta }_{1}\cdot {p}_{1}$$

2 $${{PCR}}_{2}={{N}_{{\rm{in}}}\cdot \left(1-\gamma -{p}_{1}\right)\cdot \eta }_{2}\cdot {p}_{2}$$

For simplification, the re-absorption of the reflected photon at D2 was a small value and not included in Euq.2. Waveguide loss was also not included since it was one order of magnitude lower than $\gamma$. Equ.1 and Equ.2 suggest that although ${{PCR}}_{1}$ and ${{PCR}}_{2}$ depend on the incident photon number ${N}_{\rm{in}}$ linearly, their ratio does not. The ratio is

3 $$r=\frac{{{PCR}}_{2}}{{{PCR}}_{1}}=\frac{{\left(1-{\gamma -p}_{1}\right)\cdot \eta }_{2}\cdot {p}_{2}}{{\eta }_{1}\cdot {p}_{1}}$$

Because the two detectors were fabricated on the same chip and post selected, it was reasonable to assume an identical case where ${\eta }_{1}={\eta }_{2}={\eta }_{0}$ and ${p}_{1}={p}_{2}={p}_{0}$. Therefore, ${p}_{0}=1-r-\gamma$ gives the identical absorption. To eliminate photon counting noise, counting rates were measured with an integration time of 0.1 second and repeated for 2000 times. By turning off the laser output, system dark counts (*SDCR*) were measured in the same way. Distributions of *SDCR* and the total counting rates *TCR* are shown in Fig. 4c, d. The *PCR* values were obtained by subtracting the mean value of the *SDCR* from the *TCR* when the laser was on. By using a Gaussian fitting, the mean values for *PCR*_1_ and *PCR*_2_ are 33893 and 665, from which *r* can be calculated as $r=1.96 \%$ and the identical absorption was ${p}_{0}=1-r-\gamma =97.82 \%$. By taking ${\eta }_{0}=99.99 \%$ and $\gamma =0.22 \%$, the identical *ODE* for the D1 is ${OD}{E}_{{\rm{i}}}^{1}={\eta }_{0}\cdot {p}_{0}=$97.81$\%$. For two detectors, ${{ODE}}_{{\rm{i}}}^{{\rm{sum}}}={\eta }_{0}\cdot {p}_{0}+(1-\gamma -{p}_{0})\cdot {\eta }_{0}\cdot {p}_{0}=\,$99.73$\%$, showing well consistency with room temperature measurement results ${{ODE}}_{{\rm{sum}}}=99.73 \%$.

## Discussion

In conclusion, we have demonstrated a hybrid integration of comb nanowire single photon detectors on photonic waveguides for achieving near-unity detection efficiency. Moreover, to evaluate such high detection efficiency by avoiding uncertainty during the calibration of incident photon number, two comb nanowire detectors were integrated on one waveguide, forming a self-calibration setup. Both room-temperature measurement of the absorption and cryogenic photon counting measurement showed consist results, convincing that the total detection efficiency from the two detectors can surpass 99% on a silicon waveguide.

During the entire calculation, reflection loss $\gamma$ was included of which the value was based on numerical simulation. Direct measuring $\gamma$ was challenging in our current setup since the reflectance and loss of the grating coupler were much higher than $\gamma$. $\gamma$ could be measured by placing a third detector on the reflection arm of a two-port beam splitter. However, the splitter loss and its own reflection should be controlled in a small value so that $\gamma$ can be manifested, which poses significant challenges for fabrication. $\gamma$ can also be reduced by introducing optical structures on waveguide, such as photonic crystal cavities^36,37^, to matching the mode difference. Alternatively, further increasing *ODE* to surpass 99.9% is expected on low loss and low reflection LiNbO_3_ waveguide, which is compatible to our transfer process.

It is worth noting that such high *ODE* will be beyond the accuracy of commercial power meters, which is why we chose the two-comb-nanowires setup for calibration. More rigorous calibration requires metrology innovation. A fully integrated quantum photonic chip with a deterministic photon source and detectors of near-unity detection efficiency may be one of the solutions, which is also the vision that IQP researchers are pursuing. The hybrid integration method and on-chip calibration method could make this possible. Moreover, the photon-number-resolving capability holds particular significance for continuous-variable quantum computing^38^. The hybrid integration approach maintains full compatibility with established PNR-SNSPD techniques^39–42^, thereby enabling the simultaneous achievement of high on-chip detection efficiency and photon-number resolution capability.

## Materials and methods

### SNSPD membranes fabrication

SNSPDs were fabricated on double-polished thermal oxide silicon wafers, where the oxide layer thickness is 256 nm and the silicon layer thickness is 350 μm. A 5 nm thick superconducting film (NbN) was deposited by magnetron sputtering. The photoresist involved LOR 10B (4000 rpm, 150 °C bake for 5 min) and AZ1500 (4000 rpm, 90 °C bake for 2 min). The resist stack was then patterned via laser direct writing (exposure dose: 170 mJ/cm²) and developed in orthogonal developer for 17 s to form the liftoff mask. Subsequently, 10 s of Ti and 100 s of Au were deposited by magnetron sputtering, followed by 5 min of ultrasonic-assisted liftoff in acetone. This bilayer resist liftoff process ensured excellent edge definition. Next, e-beam resist was spin-coated. Nanowire patterns were exposed by e-beam lithography. After exposure, the chip was developed in a 1:3 mixture of MIBK and IPA for 90 seconds, and the rinsed in IPA for 60 seconds. Reactive ion etching (RIE) in ${{\rm{SF}}}_{6}$ and ${{\rm{CF}}}_{4}$ was used to etch the nanowire. A second photolithography was conducted to remove the area outside of the comb nanowire structure where the waveguide was covered below.

After completing the fabrication of the comb nanowire detectors, the next steps were for etching the entire device into a membrane. Photoresist was spin-coated and heated at 90 °C for 2 min. Etching windows were patterned by using laser direct writing. After developing, the chip was soaked in BOE solution for 60 s to remove the 256 nm thick silicon dioxide layer. Then, the bottom silicon was etched by anisotropic SF₆-based RIE (Reactive Ion Etching). To move the residual photoresist, a multi-step cleaning procedure was adopted: the bulk photoresist was first dissolved in acetone, followed by NMP cleaning in an 80 °C water bath to further reduce residues, and finally an 8 s oxygen plasma treatment was applied to eliminate remaining organics and strengthen the surface energy. The brief plasma exposure duration ensured that the SNSPD sustained no measurable structural or functional degradation. It should be noted that the membrane devices are fragile; therefore, aggressive cleaning methods such as gas gun or ultrasonic bath were avoided to prevent damage to the supporting structures.

### Silicon waveguide fabrication

The silicon waveguide was fabricated on an 8-inch SOI (silicon-on-insulator) wafer with 220 nm-thick top silicon and 3-micrometer-thick buried oxide. A layer of photoresist was the first spin-coated, the 248 nm DUV (deep ultraviolet) lithography was adopted to define the circuit patterns on the photoresist. Double inductively coupled plasma (ICP) etching processes were applied to transfer the patterns from the photoresist layer to the silicon layer, forming waveguides and circuits. Deep etching waveguides with an etched depth of 220 nm were used for integrating with SNSPD. Shallow etching waveguides with an etched depth of 70 nm were used for the waveguide crossers and grating couplers.

## Supplementary information

Supplementary Information for Surpassing 99% detection efficiency by cascading two superconducting nanowires on one waveguide with self-calibration

## Data Availability

Data underlying the results presented in this paper are available.
