# Supplementary material for: Surpassing 99% detection efficiency by cascading two superconducting nanowires on one waveguide with self-calibration
Source: Light Sci Appl. 2025 Oct 17;14:369. doi: 10.1038/s41377-025-02031-5 (PMC12533016; doi:10.1038/s41377-025-02031-5)
Supplement: Supplementary file 1 — Supplementary Information for Surpassing 99% detection efficiency by cascading two superconducting nanowires on one waveguide with self-calibration [file 41377_2025_2031_MOESM1_ESM.pdf]

Supplementary Information for

Surpassing 99% detection efficiency by cascading two superconducting nanowires on one waveguide with self-calibration

Author Information

Zhen-Guo Li[1,2†], Jun Mao[3†], Yi-Jing Zhou[1†], Jia-Wei Guo[1], Shi Chen[1], Hao Hao[1], Yang-Hui Huang[1], Sai-Ying Ru[1], Nai-Tao Liu[1], Zhen Liu[1], Jie Deng[1], Fan Yang[1], Xue-Cou Tu[1,4], La-Bao Zhang[1,4], Xiao-Qing Jia[1,4], Jian Chen[1,2], Lin Kang[1,4], Jianwei Wang[3,4,5*], Qing-Yuan Zhao[1,2*], Qihuang Gong[3,4,5], Pei-Heng Wu[1,4]

[†]These authors contribute equally.

[1] Research Institute of Superconductor Electronics (RISE), School of Electronic Science and Engineering, Nanjing University, Nanjing, 210023, China

[2] Purple Mountain Laboratory, Nanjing 211111, China

[3] State Key Laboratory for Mesoscopic Physics, School of Physics, Peking University, Beijing, 100871, China

[4] Hefei National Laboratory, Hefei, Anhui 230088, China

[5] Frontiers Science Center for Nano-optoelectronics & Collaborative Innovation Center of Quantum Matter, Peking University, Beijing, 100871, China

[*] jianwei.wang@pku.edu.cn

[*] qyzhao@nju.edu.cn

Supplementary Note 1: Mode simulations on different waveguides integrated with SNSPDs

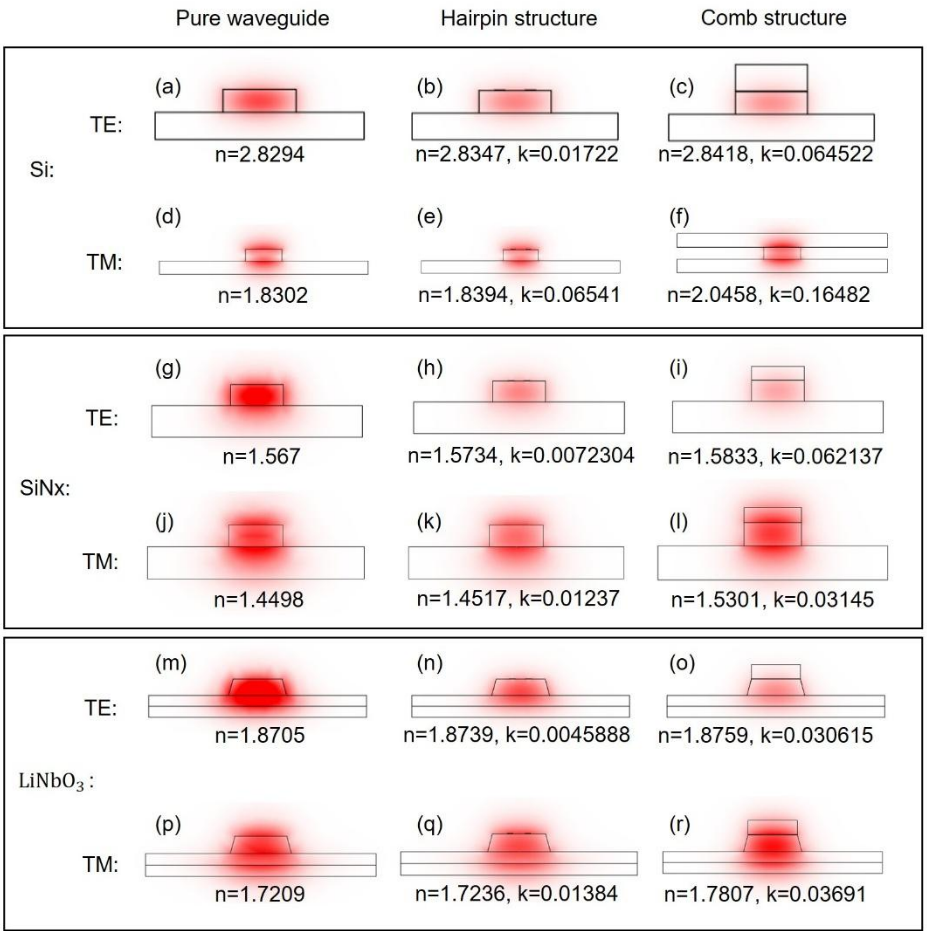

Fig.S1. TE and TM mode simulations on different waveguides (Si, $LiNbO_3$ and SiN_x) integrated with SNSPDs.

The complex mode index in an optical waveguide is used to describe the propagation characteristics of a mode, including both real and imaginary components. The real part of the complex mode index represents the phase velocity of the mode, while the imaginary part accounts for loss or gain within the waveguide. This complex index is essential for analyzing how modes propagate, especially in waveguides with dissipative or amplifying properties.

Frequency domain finite element solvers (COMSOL, Inc.) are used for numerical simulations. In the simulation, the refractive index indices of waveguide materials are $n_{\mathrm{Si}} = 3.4488$, $n_{\mathrm{SiO_2}} = 1.444$, $n_{\mathrm{NbN}} = 4.905 + i4.293$[1], $n_{\mathrm{LiNbO_3}} = 2.21$, and $n_{\mathrm{SiN_x}} = 1.996$. A two-dimensional model is established for finite element analysis to obtain the mode index of waveguide-integrated SNSPDs through modal

analysis. Mode profiles and the corresponding mode indices are shown in Fig.S1.

Supplementary Note 2: Calculation of the reflection and transmission coefficients of a comb-like nanowire using the transfer matrix method.

For the comb-structured SNSPD, its periodic structure is along the waveguide transmission direction, where each period consists of the integrated SNSPD part and the empty waveguide part, which is shown in Fig.S2a. When photons pass through a given period, they first encounter a reflection and transmission at the interface I_1 with the transmission matrix $I_1 = \frac{1}{t_1}\begin{bmatrix} 1 & r_1 \\ r_1 & 1 \end{bmatrix}$, where t_1 and r_1 represent the transmission and reflection coefficients at the interface I_1 respectively. Then, within the medium P_1, transmission and absorption occur, and the transmission matrix for this region is given by $P_1 = \begin{bmatrix} e^{-ik_{z1}d_1} & 0 \\ 0 & e^{ik_{z1}d_1} \end{bmatrix}$, where $k_{z1} = \frac{2\pi(n_1 - ik_1)}{\lambda}$, with n_1 and k_1 being the real and imaginary parts of the refractive index， and d_1 being the width of the nanowire. Next, reflection and transmission occur at the interface I_2, with the transmission matrix $I_2 = \frac{1}{t_2}\begin{bmatrix} 1 & r_2 \\ r_2 & 1 \end{bmatrix}$, where t_2 and r_2 are the transmission and reflection coefficients at the interface I_2. Finally, transmission and losses occur in the medium P_2, with the transmission matrix $P_2 = \begin{bmatrix} e^{-ik_{z2}d_2} & 0 \\ 0 & e^{ik_{z2}d_2} \end{bmatrix}$, where $k_{z2} = \frac{2\pi(n_2 - ik_2)}{\lambda}$, with n_2 and k_2 being the real and imaginary parts of the refractive index.， and d_2 being the gap between the nanowires. The transmission matrix for each period is given by $M_n = P_2 \cdot I_2 \cdot P_1 \cdot I_1$. Assuming the number of nanowires is m, the total transmission matrix is given by $M = M_{\mathrm{m}} \cdot M_{\mathrm{m-1}} \cdots\cdots M_2 \cdot M_1$. Based on the total transmission matrix M， the total reflection coefficient γ is given by $\gamma = \left|\frac{M_{21}}{M_{11}}\right|^2$, the total transmission coefficient T is given by $T = \frac{1}{{M_{11}}^2}$, where M_{11} is the element in the first row and first column of the matrix M, and M_{21} is the element in the second row and first column.

To verify the correctness of the transfer matrix method, we compared it with the results of 3-D simulation. Due to the limitations of computational power, we only simulated the cases with 1 to 20 nanowires, corresponding to waveguide lengths ranging from 0 to 6 μm. The number of nanowires, num, and the waveguide length, L, satisfy the equation $L = 0.3 \times num$ (μm). As shown in Fig.S2b, c, d, the results of the transfer matrix method are in excellent agreement with the 3-D simulation results.

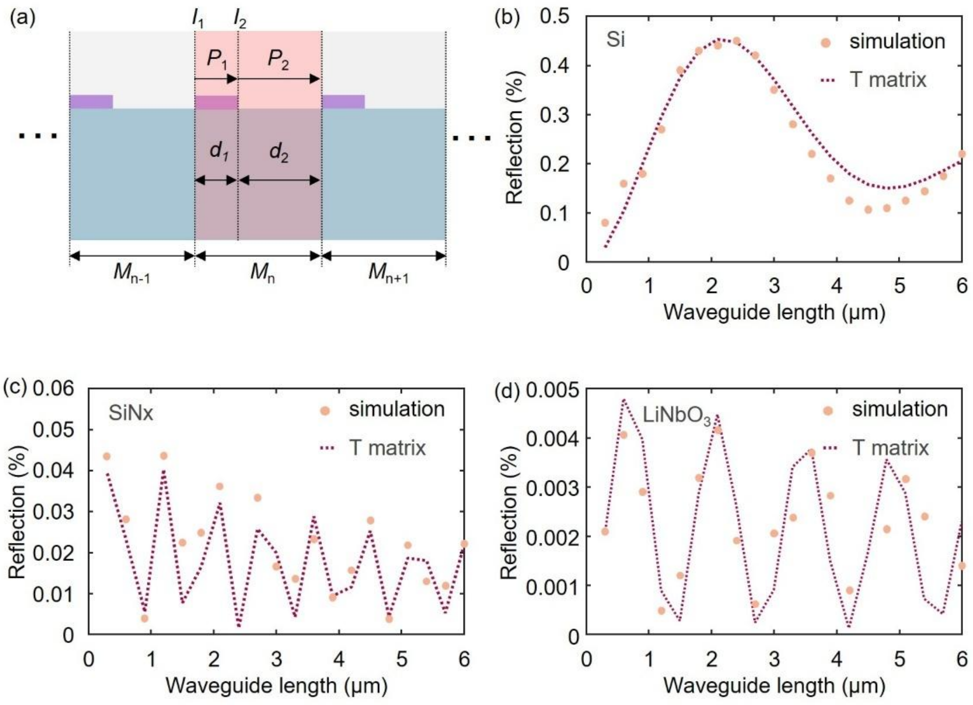

Fig.S2. Simulation in transfer matrix method (a) The cross-sectional diagram of the comb nanowires along the optical waveguide propagation direction. The reflection coefficient of Si (b), SiN_x (c), and $LiNbO_3$ (d) waveguide integrated comb nanowire calculated by 3-D numerical simulation (red dashed line) and the transfer matrix method (orange dots).

Supplementary Note 3: Calculation of waveguide losses and nanowire absorption coefficients.

Typically, we consider the absorption rate as $p = 1 - \gamma - T$. This result does not take waveguide losses into account. Despite the current mainstream waveguides achieving low loss levels, these losses cannot be ignored when pursuing unity detection efficiency. We need to separate the waveguide losses from the absorption of the nanowire itself. According to Beer-Lambert law, when light with initial intensity I_0 propagates a distance L along a fixed direction, the remaining intensity $I(L)$ can be calculated by:

$$I(L) = I_0 e^{-(\alpha f + \beta)L} \tag{1}$$

Here α is the absorption factor of SNSPD, defined as: $\alpha = \frac{4\pi k}{\lambda}$, β is the attenuation factor of waveguides, k is the imaginary part of the refractive index of the transmission medium, λ is the wavelength of light, f is the filling factor of the meandered SNSPD in a comb structure. In a hairpin structure, f is 1. Therefore, according to the imaginary part of the refractive index k, simulated in Fig.S1, we can deduce the

absorption factor α. The absorption of light by the SNSPD at position L, $A(L)$, satisfies: $\frac{dA(L)}{dL} = -\alpha f I(L)$. Solving this, we get: $A(L) = \frac{\alpha f}{\alpha f+\beta} I_0\left(1 - e^{-(\alpha f+\beta)L}\right)$. Similarly, for the waveguide attenuation $B(L)$, it satisfies the equation: $\frac{dB(L)}{dL} = -\beta I(L)$. Solving for it, we get $B(L) = \frac{\beta}{\alpha f+\beta} I_0\left(1 - e^{-(\alpha f+\beta)L}\right)$. Combining the transmission and reflection coefficients obtained using the transfer matrix method, we can calculate the actual absorption rate p as:

$$p = \frac{\alpha f}{\alpha f + \beta}(1 - \gamma - T) \tag{2}$$

Supplementary Note 4: Calculation of the waveguide attenuation factor *β*.

During the simulation, we typically assume the imaginary part of the waveguide's refractive index is zero, thus the waveguide loss cannot be determined through the 2D finite element simulation. Nevertheless, we can back-calculate the waveguide's attenuation factors β based on actual test results. The typical levels of attenuation coefficients B for several mainstream waveguides are investigated for calculation the net absorption by nanowires, which is shown in Table S1.

Table S1: The attenuation coefficients of different waveguides.

	Si waveguide	SiN_x waveguide	$LiNbO_3$ waveguide
B ($dB{\cdot}cm^{-1}$)	0.1~3[2]<br>-1.4278 (experimental data)	-0.024[3]	-0.15[4]
β (μm^{-1})	3.29×10^{-5}	5.53×10^{-7}	3.45×10^{-6}

According to Beer-lambert law, we can derive the attenuation formula for light in the optical waveguide: $I(L) = I_0 e^{-\beta L}$ where β is the attenuation factors of waveguides. However, the attenuation coefficient B represents the loss of the waveguide per unit length, defined as:

$$B = \frac{10 \times log_{10}\frac{I(L)}{I_0}}{L} = -10 \times log_{10}e \times \beta \tag{3}$$

Similarly, we can also determine the relationship between the absorption coefficient and the absorption factor:

$$A = -10 \times log_{10}\, e \times \alpha \tag{4}$$

Taking silicon waveguides as an example, the attenuation coefficient of the silicon waveguide B_{si} is -1.4278 dB cm^{-1} at a wavelength of 1.55 micrometers, as shown in Fig.S3. The attenuation factor of silicon waveguide is: $\beta_{\text{si}} = \frac{B_{\text{si}}}{-10 \times log_{10} e} = 0.329\ \text{cm}^{-1}$. Considering that both the waveguide length and the nanowire span are on the micrometer scale, we will also standardize the dimension of β_{si} to micrometers, $\beta_{\text{si}} = 3.29 \times 10^{-5}\ \mu\text{m}^{-1}$. Using the same method, we can determine the attenuation factors β for different waveguide platforms.

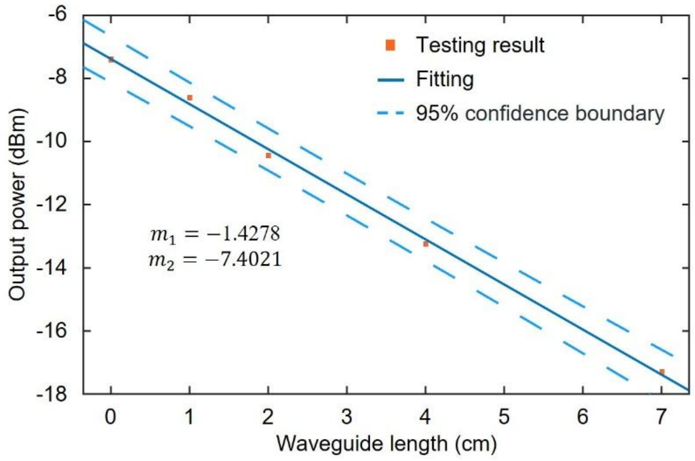

Fig.S3. Attenuation curve of the silicon waveguide. We input an optical power of 4 dBm, coupled into silicon waveguides of lengths 0 cm, 1 cm, 2 cm, 4 cm, and 7 cm, and measured the output optical power. The function $f(x) = m_1 x + m_2$ is used for fitting, where m_1 represents the attenuation coefficient of the silicon waveguide, $\frac{m_2 - 4}{2}$ represents the attenuation of the grating coupler. With a 95% confidence interval, the average attenuation coefficient is -1.4278 dB·cm^{-1}, with an upper bound of -1.3189 dB cm^{-1} and a lower bound of -1.5366 dB cm^{-1}. The average attenuation of the grating coupler is -5.7011 dB, which determines the coupling efficiency of 26.91 % at room temperature.

Supplementary Note 5: The absorption is insensitive to tilt under this resolution

In the transfer process, the alignment accuracy may affect the final absorption rate. Since the width of our detection area is larger than the waveguide width, offsets within 1 μm do not affect the absorption

rate. Another factor affecting the absorption rate is slight rotation, as shown in Fig.S4a. The detection area has a width of 6μm and a length of 30μm, while the waveguide width is 700nm. Therefore, the maximum offset angle cannot exceed $arctan(\frac{6-0.7}{30}) = 10.0210°$. To investigate this, we conducted systematic 3D simulations using COMSOL to quantitatively analyze the effect of small-angle tilt on absorption. The simulation model exactly replicates the geometry shown in Fig.1e of the main text. To optimize computational efficiency, we conducted the angular tolerance study using a reduced 15-nanowire 3D model (rather than simulating the full 100-nanowire/30 μm device) to evaluate the impact of rotation angles from 0 to 10° on the absorption rate. The simulation result is shown in Fig.S4b, indicating that the loss in absorption rate is almost negligible.

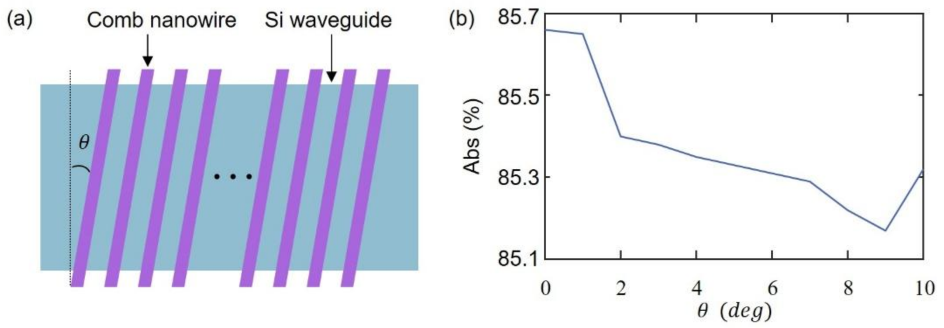

Fig.S4. Simulation of the impact of small-angle tilt on the absorption rate. (a) A top-down view of a waveguide-integrated comb nanowire. (b) The curve showing the relationship between absorption rate and tilt.

Supplementary Note 6: Calculation of detection efficiency loss due to the corner effect in a hairpin nanowire

At the corners of the hairpin SNSPD, the current density in some areas is much lower than that in the nanowire sections. When the bias current in the nanowire reaches the maximum, the current density at the corners remains very low, resulting in near-zero quantum efficiency and almost negligible photon detection capability. Therefore, these regions are referred to as a dead area. Photons must pass through the dead area before reaching the nanowire region; the dead area absorbs photons but does not generate pulses, which can be considered as a loss. In order to quantitatively calculate the current crowding effect at the corner, we simulated the current density distribution at the corner of a nanowire, as

shown in Fig.1c, where the boundary of the dead zone is only qualitatively displayed. The magnitude of the bias current is related to the quantum efficiency, and it can be quantitatively described by: $IDE = \frac{1}{2} erfc(\frac{I_{co}-I_B}{\Delta I_B})$ based on the Fano fluctuation model. Therefore, with the current density distribution, the quantum efficiency distribution is obtained. By integrating the *IDE* over the nanowire region, the equivalent effective area can be obtained. The dead zone area is calculated by subtracting the effective area from the total area of the nanowire region. By dividing the dead zone area by the width of the incident cross-section of the nanowire, the dead zone equivalent length is obtained:

$$l_{\text{equal}} = \frac{\iint ds - \iint \frac{1}{2} erfc\left(\frac{I_{co} - J}{\Delta I_B}\right) ds}{w} \tag{5}$$

where $w = 400$ μm is the width of the dead area, J is the current density distribution. To minimize such loss, waveguide integrated detectors have to tolerate some current crowding effect with using narrow corners, such as using a semi-circular corner as shown in Fig.1c. Even so, the equivalent length of the dead area reach 48.974 nm. Thus, the absorption rate of the dead area Abs_{dead} is given by

$$Abs_{\text{dead}} = e^{-(\alpha+\beta) l_{\text{equal}}} \tag{6}$$

Here, β is given in Supplementary Table 1, $\alpha = \frac{4\pi k'}{\lambda}$. To determine the value of k', we performed a two-dimensional finite element simulation with a nanowire width of 400 nm and a thickness of 5 nm at the cross-section. The simulation results are shown in Fig.S5.

Fig.1d gives the calculation of the corner loss for a hairpin structure of 100 nm wide nanowire and 200 nm wide gap. For an optimal corner, the loss is 5.3 % for Si waveguide, 3.0 % for SiN_x waveguide and 1.5 % for $LiNbO_3$ waveguide. For a semi-circular narrow corner, the loss is 1.9 % for Si waveguide, 1.0 % for SiN_x waveguide and 0.5 % for $LiNbO_3$ waveguide. The semi-circular corner has a strong current-crowding effect. The maximum bias current is suppressed to 69.4 % of the critical current.

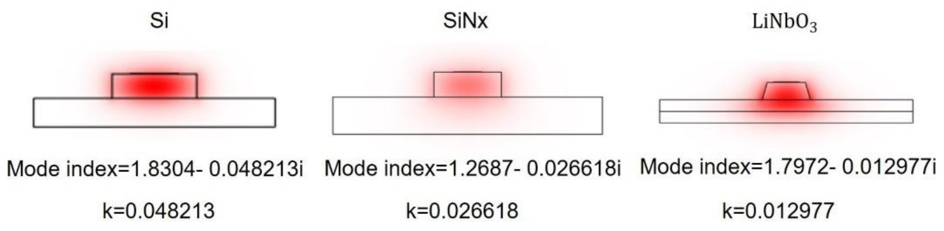

Fig.S5. Two-dimensional finite element simulation results of the cross-section at the corner.

Supplementary Note 7: Cryogenic characterization setup

The cryogenic characterization experiments were conducted in a customized 1.5 K cryogenic probe station system, which includes a set of four-axis movable optical fiber probes and a set of three-axis movable electrical probes, shown in Fig. S6b. A pulsed laser with a wavelength of 1550 nm was emitted and passed through two variable attenuators and a polarization controller before entering the low-temperature chamber. The light was then coupled to the waveguide chip's grating coupler through the optical fiber probes, while the SNSPD was biased via electrical probes connected to a tunable constant current source. The response pulses were amplified and fed into a pulse counter. The test system setting is shown in Fig.S6a. During the test, we adjusted the optical attenuator to ensure that the SNSPD's light count was less than one-tenth of the pulsed laser's synchronization signal, approximating single-photon incidence. We measured the PCR curve for D1 and D2, as well as the distribution of C1 and C2 with the bias current fixed, and repeated the measurements 2000 times.

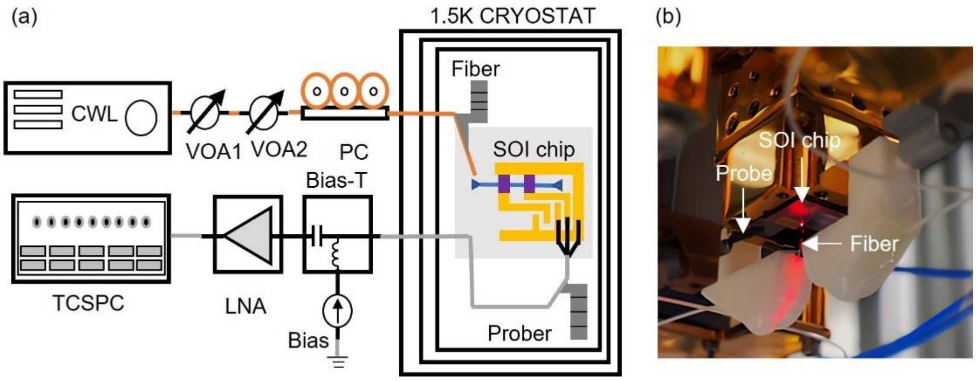

Fig.S6. Cryogenic characterization setup. (a) Cryogenic testing system block diagram. (b) Probe

structure inside the cryostat.

Supplementary Note 8: Characterization of system detection efficiency

The test system block diagram is shown in Fig.S6. We calibrated the laser output power to 7.95 dBm using an optical power meter. Then, we calibrated the losses of the attenuator, polarizer, and related fiber insertion losses, ultimately determining that the actual optical power entering the cryostat was -116.41 dBm, which corresponds to an incident optical flux of 17,822 cps. Fig.S7a provides a visual overview of the incident photon calculation procedure. At a bias current of 5.5 μA, the measured photon count rate is 2,540 cps. As shown in Fig.S7b, the system detection efficiency is calculated by: $SDE = \frac{PCR}{N_{\text{in}}} = 14.3\%$, which includes the coupling loss of the grating coupler and the insertion loss of the fiber entering the cavity. Due to the lack of high-precision six-axis positioners in the cryostat, the optimal coupling efficiency could not be achieved, resulting in a lower system detection efficiency test value.

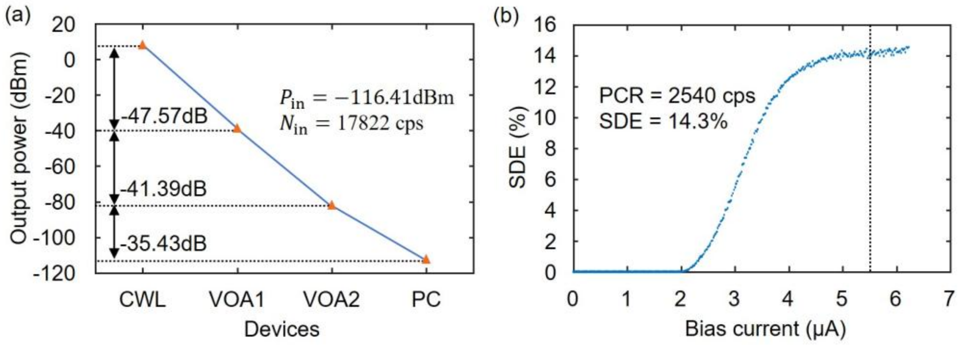

Fig.S7. Characterization of system detection efficiency. (a) Attenuation of different optical device. (b) System detection efficiency curve.

Supplementary Note 9: Calculation of quantum efficiency

As established in the reference work[5], the relationship between photon count rate PCR and bias current I_b follows:

$$PCR = \frac{a}{2}\operatorname{erfc}\left(\frac{I_{\text{CO}} - I_{\text{B}}}{\Delta I_{\text{B}}}\right) \tag{6}$$

where a is the photon number absorbed by SNSPD, I_{CO} is the cutoff current. Taking D1 as an example, we fitted the photon count rate using Equ.6 within a 95% confidence interval. The fitting result is shown in Table S2. The quantum efficiency IDE is obtained by dividing the photon count rate *PCR* by the absorbed photon number a:

$$IDE = \frac{1}{2}\mathrm{erfc}\left(\frac{I_{\mathrm{CO}} - I_{\mathrm{B}}}{\Delta I_{\mathrm{B}}}\right) \quad (7)$$

For device D1, operating at a bias current of $I_{\mathrm{B}} = 5.5$ μA, substitution of I_{B}, I_{CO}, and ΔI_{B} into Equa.7 yields a quantum efficiency of 99.99%.

Table S2: Parameter bounds at 95% confidence interval and quantum efficiency at 5.5 μA bias current.

	I_{CO}	ΔI_{B}	a	IDE (%)
min	3.1800	0.8396	33084	99.99534
center	3.1869	0.8528	33185	99.99374
max	3.1938	0.8660	33287	99.99171

Supplementary Note10: Characterization of the yield rate.

We prepared 219 SNSPD membranes and used an automated cryogenic probe station system shown in Fig.S6 for characterization. The distribution of superconducting critical current is shown in Fig.S8a. We characterized their PCR, normalized the bias current and photon counts, which is shown in Fig.S8b. The normalized PCR at $0.8I_{\mathrm{c}}$ was taken, with values above 80% considered as saturated products, between 40% and 80% as constricted products, and below 40% as weak products. Out of these, 132 were classified as saturated products, resulting in a yield of 60.27%, 41 were classified as constricted products, resulting in a yield of 79%.

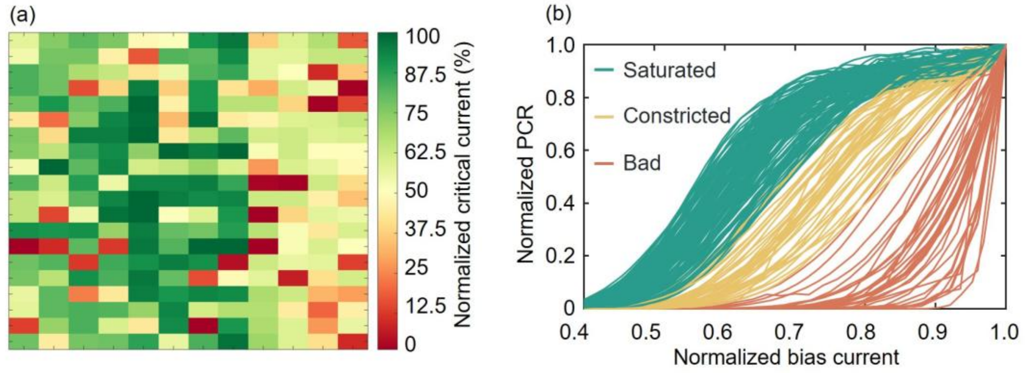

Fig.S8. Performance variation among 219 membrane devices fabricated on the same chip. (a)The distribution of superconducting critical current. (b)The normalized PCR curve.

Supplementary Reference

1. Yamashita, T. et al. Low-filling-factor superconducting single photon detector with high system detection efficiency. *Opt. Express* **21**, 27177 (2013).
2. Marpaung, D. et al. Integrated microwave photonics. *Nat. Photon.* **13**, 80–90 (2019).
3. Liu, J. et al. High-yield, wafer-scale fabrication of ultralow-loss, dispersion-engineered silicon nitride photonic circuits. *Nat. Commun.* **12**, 1–9 (2021).
4. Ye, Z. et al. Fabrication and Characterization of Wafer-Scale Thin-Film Lithium Niobate Waveguides. *J. Synth. Cryst.* **53**, 426–433 (2024).
5. Kozorezov, A. G. et al. Fano fluctuations in superconducting-nanowire single-photon detectors. *Physical Review B* **96**, 054507 (2017).
